# Double-targeting CDCA8 and E2F1 inhibits the growth and migration of malignant glioma

**DOI:** 10.1038/s41419-021-03405-4

**Published:** 2021-02-01

**Authors:** Xiaoxiong Wang, Heping Wang, Jiajun Xu, Xu Hou, Haoqiang Zhan, Yunbo Zhen

**Affiliations:** 1 Department of Neurosurgery, The First Affiliated Hospital of Harbin Medical University, No. 23 Youzheng Street, Nangang District, 150001 Harbin, Heilongjiang Province People’s Republic of China; 2 Institute of Brain Science, Harbin Medical University, No. 23 Youzheng Street, Nangang District, 150001 Harbin, Heilongjiang Province People’s Republic of China; 3 Department of Neurosurgery, TongJi Hospital of TongJi Medical College, Huazhong University of Science and Technology, No.1095 Jie Fang Avenue, Hankou, Wuhan, 430030 People’s Republic of China; 4 Department of Neurosurgery, The Sixth Affiliated Hospital of Sun Yat-sen University, No. 26 Erheng Road, Yuan Village, Tianhe District, Guangzhou, People’s Republic of China

**Keywords:** Cancer, Cancer

## Abstract

High-grade glioma is the most common and aggressive primary brain tumor in adults with poor therapeutic efficiency and survival prognosis. Cell division cycle associated 8 (CDCA8) has been well known as a cell cycle regulator and tumor promotor in various malignant tumors. However, its biological role in glioma still remains unclear. Our results showed that high level of CDCA8 was significantly correlated with advanced WHO grade and poor overall survival and disease-free survival prognosis. In vitro and in vivo investigations demonstrated that CDCA8 promoted the glioma malignancy by promoting cell proliferation, cell migration, and inhibiting cell apoptosis. Moreover, we found its synergetic biological protein—E2F1 by the gene microarray chip. In this study, we revealed that CDCA8 synergized with E2F1 facilitated the proliferation and migration of glioma. In conclusion, our study provides a novel promising therapeutic targets and prognostic biomarkers for malignant glioma treatment.

## Introduction

High-grade glioma is the most common malignant primary brain tumor. Despite current advances in treatment for high-grade glioma, the overall prognosis remains poor, and long-term survival is rare^1,2^. To date, the multimodal therapy for malignant gliomas incorporating surgery, radiotherapy, systemic therapy (chemotherapy, targeted therapy), and supportive care improved the therapeutic effect of single therapy^3,4^. However, due to the clinical characteristics of invasive growth, malignant glioma cells migrate outward along the brain white matter, para-vascular spaces, or nerve fibers, making it impossible to be completely removed. Even if the patient undertook the comprehensively therapeutic strategy, these tumor seeds hidden in the cerebral microenvironment niches may still cause rapid recurrence, which is the major factor for poor survival of patients with malignant gliomas^5,6^. Approximately 50% of postoperative gliomas recur with more aggressively malignant biological behaviors and rapidly deteriorative circumstances. Current studies have shown that hypoxic microenvironment, key oncogenetic and metabolic pathways are involved in the initiation and aggression of malignant gliomas^6–8^. The era of precision oncology renders much promises in developing more efficient therapies to confront this aggressive disease. Therefore, the elaboration of the underlying molecular mechanism will provide potential therapeutic targets and prognostic biomarkers for novel glioma therapeutic strategies.

Chromosomal passenger complex (CPC) is the important regulatory structure involved in cell proliferation and division^9^. CDCA8 (also known as Borealin/DasraB) protein encoded by human cell division cycle associated 8 gene is another major component in the CPC, except INCENP, Survivin, and Aurora B^10^. *CDCA8* as cell cycle regulatory gene plays a key role in recruiting CPC to centromeres, correcting kinetochore binding errors, and stabilizing double-click spindles^11^. In recent years, increasing studies have shown that overexpressed CPC component proteins are responsible for the dysfunction of chromosomal passenger complexes, abnormal cell division, and aneuploidy, thereby accelerating the tumorigenesis and malignancy^9,10,12^. The components of CPC, Survivin, and Aurora B, are overexpressed in various types of cancers, while another two components, CDCA8 and INCENP, are rarely studied in tumors^13–15^. The analysis of CDCA8 expression showed that it was highly expressed in tumor cells and undifferentiated human embryonic stem cells, but no or weakly expressed in normal tissue cells^16,17^. Notably, the biological function of CDCA8 has not been extensively investigated in glioma.

In the present study, we demonstrated the prognostic role of CDCA8 in high-grade glioma. And we further screened target genes of CDCA8 and investigated the potential role of CDCA8 synergized with the transcription factor E2F1 in glioma cell proliferation and migration, indicating the underlying molecular mechanism of CDCA8/E2F1-associated tumor suppression in glioma treatment.

## Materials and methods

### Cell culture

Human glioma cell lines U251 and SHG-44 were obtained from the Hangzhou BeNa Technology. SHG-44 and U251 cells were maintained in 90% DMEM medium (Gibco) supplemented with 10% FBS. All cells were grown in an incubator at 37 °C with 5% CO_2_.

### Immunohistochemistry analysis (IHC)

Glioma tissue and adjacent normal tissue microarray (Cat. #HBraG180Su01, Shanghai Outdo Biotech Company) was applied for IHC analysis. Before IHC assay, the microarrays were baked at 65 °C for 30 min. Next, microarrays were dewaxed in xylene and hydrated in ethanol with different concentrations. Citrate buffer was used for antigen repairing at 180 °C for 5 min. After blocked with 3% H_2_O_2_, anti-CDCA8 was incubated with the sections at 4 °C overnight. Secondary antibody was added and incubated for 2 h at room temperature. Finally, the tissue microarrays were stained with diaminobenzidine and analyzed using CaseViewer 2.0 and Image Scope. The IHC scoring of specimens was determined by the staining intensity and extent scores, which graded as 0 (0%), 1 (1–25%), 2 (26–50%), 3 (51–75%), or 4 (76–100%). Antibodies used were shown in Table S1.

### Plasmid construction, lentivirus infection, and transfection

shRNA sequences of human gene CDCA8 and E2F1 were designed at Shanghai Yibeirui Biomedical Science and Technology Co., Ltd (sequences for CDCA8 and E2F1 were detailed in Table S2). Target shRNA sequences were inserted into BR-V-108 vectors, and then transformed into *E. coli* competent cells (Tiangen) for plasmid extraction using EndoFree maxi plasmid kit (Tiangen). Qualified plasmids were packaged for lentivirus production using 293T cells which were co-transfected with BR-V-108, pHelper 1.0, and 2.0 Vector with Lipofectamine 2000 reagent (Thermo Fisher Scientific). Lentiviral vector containing amplification sequence of CDCA8 was also constructed for overexpression. Human glioma cell lines stably expressing CDCA8 and E2F1 protein were established by transfecting the BR-V-108-CDCA8 or BR-V-108-E2F1lentivirus into SHG-44 and U251 cells at 80% confluence using Lipofectamine 2000 reagent (Thermo Fisher Scientific). After 72 h, cell infection efficiency was evaluated by microscopic fluorescence. Lentivirus transfected cells were prepared for the next experiment.

### RNA extraction and RT-qPCR

Lentivirus transfected cells at 80% confluence were harvested and total RNA was extracted using Trizol reagent (Invitrogen). The quality of total RNA was evaluated by Nanodrop 2000 spectrophotometer (Thermo Fisher Scientific) according to the manufacturer’s instructions. 2.0 μg total RNA was reverse transcribed into cDNA using Promega M-MLV. 1.0 μL cDNA was added into the reaction system for real-time PCR using SYBR Green master mix Kit (TAKARA) on the platform of Sequence Detection system (TAKARA). The related primers used were shown in Table S3. The relative quantitative analysis in gene expression, data were analyzed by the 2^−ΔΔCt^ method with GAPDH as inner control.

### Western blotting assay (WB)

Lentivirus transfected cells at 80% confluence were harvested and lysed in ice-cold lysis buffer, and the total protein collected and the protein concentration was detected by BCA Protein Assay Kit (HyClone Pierce). Equal amount proteins (20 µg) were separated by 10% SDS-PAGE. After all, proteins were transferred onto PVDF membrane, the membrane was blocked at room temperature for 1 h with 5% non-fat milk TBST solution. Then the blocked membrane was incubated with primary antibodies at 4 °C overnight and continuingly incubated with the secondary antibody for 2 h at room temperature. The outcomes were visualized by ECL plus TM Western blotting system kit (Amersham). Gray scanning was analyzed by Image J. Antibodies used in WB were detailed in Table S1.

### MTT assay

Lentivirus transfected cells in exponential growth phase were trypsinized and seeded into a 96-well plate (2000 cells/well) and cultured. In all, 20 μL of 5 mg/mL MTT solution (Sangon Biotech) was added at 1st, 2nd, 3rd, 4th, and 5th day, respectively. After reaction for 4 h, 100 μL DMSO solution was added and the absorbance values at 490 nm were measured by a microplate reader (Tecan). Cell viability was detected by MTT assay according to the manufacturer’s protocol.

### Flow cytometry assay

Apoptosis and cell cycle were detected by flow cytometry assay. Lentivirus transfected cells were inoculated in 6-well dishes with 5 mL per well (1 × 10^3^ cells/mL) in triplicate and cultured until the confluence reached 70%. Cells were collected and washed with 4 °C D-Hanks, then centrifugated (1300 rmp). Cells were resuspended with 200 μL 1× binding buffer, and stained by 10 μL Annexin V-APC (eBioscience) in the dark. For cell cycle detection, cells were dyed by PI (Sigma) staining solution, then cells were detected using Millipore flow cytometry.

### Colony formation assay

Cells in the logarithmic growth phase were trypsinized, resuspended, and seeded into 6-well plates at 600 cells per well in triplicate. After cultured for 8 days, cell clones were photographed under a fluorescence microscope (Olympus). Then all clones were fixed by 4% paraformaldehyde (SIGMA) and stained by Giemsa. Finally, clones were washed with ddH_2_O several times, dried, and photographed with a digital camera. Colony-forming rate = (colony number/inoculated cell number) × 100%.

### Wound-healing assay

Lentivirus transfected cells were seeded at 3 × 10^4^ cells per well onto a 96-well dish. Scratches were made by a 96-wounding replicator (VP scientific) across the cell layers while the cell confluence reached over 90%. Serum-free medium was used to rinse gently 2–3 times and floating cells were washed. RPMI-1640 medium with 0.5% FBS was added and cultured for 24 h and photographs were taken by a fluorescence microscope at 8 and 24 h. Cell migration rate of each group was calculated.

### Transwell assay

Transwell assay was performed by Corning Transwell Kit. First, transfected SHG-44 and U251 cells were collected, trypsinized, counted, and incubated in the upper chamber with 100 μL medium without FBS in a 24-well plate (1 × 10^6^ cells/well). Six hundred microliters medium supplemented with 30% FBS was added in the lower chamber. Cells were incubated for 48 h and then non-metastatic cells were removed with a cotton swab. Cells were fixed and stained by 4% formaldehyde and Giemsa, respectively, then the migration ability of cells was analyzed.

### Cell counting assay

Transfected cells were seeded into 96-well plates (2000 cells/well) and further cultured in DMEM supplemented with 10% FBS at 37 °C with 5% CO_2_ for 5 days. Medium was changed every three days. Celigo image cytometer (Nexcelom Bioscience) was applied for cell counting on days 1, 2, 3, 4, 5 and the data were analyzed.

### Co-immunoprecipitation (Co-IP) assay

Total protein from lentivirus transfected SHG-44 cells expressing CDCA8 were collected and Co-IP assay was applied for identifying whether CDCA8 and E2F1 interact with each other. 1.0 mg total protein was incubated with anti-DYKDDDDK Tag (binds to the same epitope as Sigma’s Anti-FLAG® M2 Antibody), anti-HA, anti-CDCA8, and anti-E2F1 at 4 °C overnight. Twenty microliters agarose beads were added and incubated at 4 °C for 2 h. After centrifugation at 2000 × *g* for 1 min, supernatant was discarded. Protein A/G beads were collected and washed twice. Next, the Protein A/G beads were denatured in the IP lysate buffer and 5× loading buffer at 100 °C boiling water for 5 min. Finally, 20 µg protein sample was subjected WB analysis described in WB assay section.

### Microarray assay

Total RNA was extracted by the RNeasy kit (Sigma). Concentration and values of A260 and A280 of total RNA were determined by Nanodrop 2000 (Thermo Fisher Scientific). RIN value was evaluated with Agilent 2100andAgilent RNA 6000 Nano Kit. RNA sequencing was performed with Affymetrix human Gene Chip PrimeView according to the manufacturer’s instruction and the outcomes were scanned by Affymetrix Scanner 3000 (Affymetrix). Gene expression in SHG-44 cells transfected with shCDCA8 or shCtrl was analyzed with PrimeView Human Gene Expression Array (Affymetrix) and the microarray raw data were generated, statistical significance assessment was accomplished using a Welch *t*-test with Benjamini–Hochberg FDR (log2 fold change >1.3 and FDR < 0.05 as significant). Significant difference genes bioinformatics analysis based on Ingenuity Pathway Analysis (IPA) (Qiagen) was executed, and |Z-score|>2 is considered meaningful.

### Human apoptosis antibody array

Detection of related genes in human apoptosis signaling pathway was performed using Human Apoptosis Antibody Array (Abcam, ab134001) that simultaneously detects 43 Human Apoptosis marker concentrations in cell and tissue lysates. According to the manufacturer’s instructions, the SHG-44 cells expressing CDCA8 were lysed and total proteins were extracted. Protein concentrations were measured by BCA Protein Assay Kit (HyClone Pierce). Antibody array ships were incubated with protein samples (0.5 mg/mL) overnight at 4 °C and continuing incubated with cocktail of biotin-conjugated antibodies overnight at 4 °C. Next incubated with labeled streptavidin for 2 h. Enhanced chemiluminescence was used for visualizing and spots gray value was analyzed by Image J.

### Tumor transplantation model

Female BALB/c nude mice were purchased from Beijing Vitalriver Experimental Animal Technology Co., Ltd and housed in a 12 h light/dark cycle controlled condition at 24 °C. 0.2 mL (4 × 10^6^ cells/mice) lentivirus transfected SHG-44 cell suspension was subcutaneously injected into 4-week-old mice (5 mice per group, group as NC vs CDCA8, NC vs shE2F1, NC (KD + OE) vs CDCA8 + shE2F1; or 10 mice per group, group as NC and KD). The lengths (*L*) and widths (*W*) of tumor was record and tumor volume later using *L* and *W* (*L* represent longest dimension and *W* means dimension perpendicular to length) and calculated as π/6 × *L* × *W*^2^. Tumor volume was calculated 2–3 times weekly. Fluorescence images were observed by IVIS Spectrum Imaging System (Perkin Elmer). At the end of animal experiment, all mice were sacrificed and tumor tissues were removed for Ki67 immunostaining. All animal experiments conformed to the European Parliament Directive (2010/63/EU) and were approved by the Institutional Animal Care and Use Committee at Harbin Medical University (No. HMUIRB-2008-06) and the Institute of Laboratory Animal Science of China (A5655-01).

### Ki67 immunostaining

For Ki67 immunostaining, tumor tissues were fixed in 4% paraformaldehyde for 24 h, and then were embedded in paraffin for cutting (4 μm). After deparaffinization, rehydration, and blocking with PBS-H_2_O_2_, all sections were incubated with Ki67 primary antibody at 4 °C overnight. After washing, sections were further incubated with the secondary antibody, antibodies were detailed in Table S1. Immunostaining was applied by Hematoxylin and Eosin (Baso, Zhuhai, Guangdong, China).

### Surgically excised specimens of glioma patients

A total of 176 glioma patients were enrolled in the Department of Neurosurgery at The First Affiliated Hospital of Harbin Medical University from 2017 to 2019. The mean age of patients was 41 years (range, 8–80 y); 62% were male. The median preoperative KPS score was 70. None of the patients received preoperative radiotherapy or chemotherapy. The tissue samples were routinely collected for histologic diagnosis in strict accordance with WHO criteria, while adjacent specimens were immediately snap-frozen in liquid nitrogen and then stored at −80 °C until further analysis. Normal brain tissues were obtained from normal adjacent tissues away from tumor tissues or nonneoplastic brain diseases and were histologically confirmed to be free of any pathological lesions. The study was approved by the Ethics committees at the Harbin Medical University, and all subjects gave informed consent.

### Statistical analysis

Data from independent experiments are shown as the means ± standard deviations (SD). Statistical analysis between two groups was performed by Student’s *t*-test (two-tailed) and among multiple groups was conducted by one-way ANOVA with SPSS version 17.0 (IBM Analytics, USA). CDCA8 expression difference between glioma tissue and adjacent normal tissue was analyzed with Rank Sum test analysis. The relationship of CDCA8 expression and tumor characteristics in glioma patients were analyzed with Mann–Whitney U analysis and Spearman Rank correlation analysis. Survival data were evaluated by using Kaplan–Meier survival analysis. A *P* value <0.05 was considered statistically significant (*).

## Results

### High expression of CDCA8 in glioma predicts poor prognosis

**Fig. 1:**
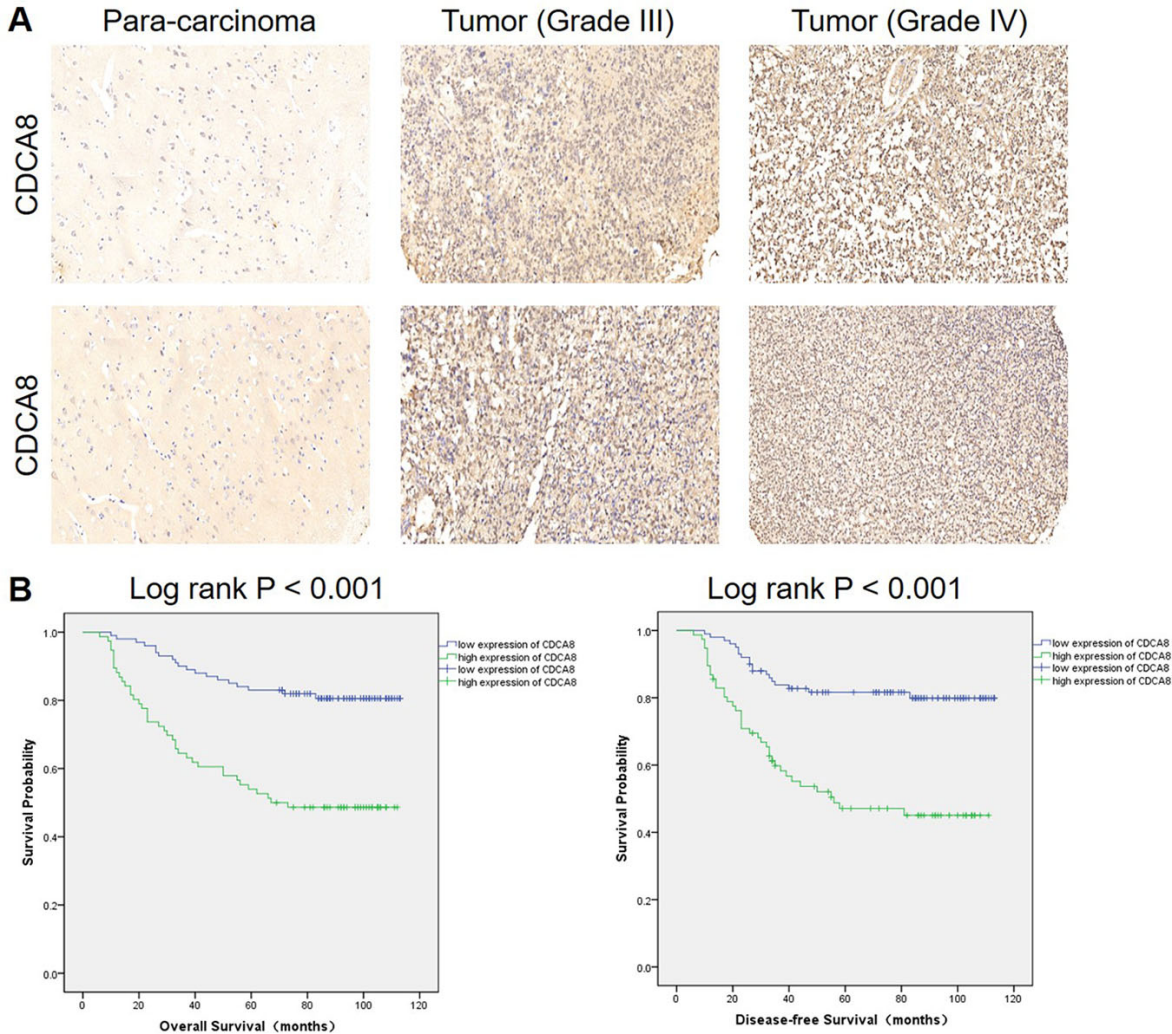
High expression of CDCA8 in glioma predicts poor prognosis. **A** CDCA8 expression in para-carcinoma tissues and glioma tissues with different tumor grade. **B** Kaplan–Meier survival analysis of CDCA8 expression in glioma patients.

To determine whether the CDCA8 expression is correlated with the malignant progression and clinical outcomes of gliomas, we detected the CDCA8 level in glioma IHC samples and evaluated the overall survival and disease-free survival outcomes by Kaplan–Meier survival analysis. As shown in Table 1 and Fig. 1A, the expression of CDCA8 was generally higher in glioma tissues. Moreover, the higher level of CDCA8 is associated with advanced WHO grade and tumor recurrence (Table 2 and Table S4). Kaplan–Meier survival analysis showed that patients with relatively higher CDCA8 expression suffer from lower overall survival rate and disease-free survival rate (Fig. 1B). These results indicate that CDCA8 may be a prognostic biomarker for glioma malignancy and poorer prognosis.

**Table 1:** Expression patterns of CDCA8 in glioma tissues and normal tissues revealed in immunohistochemistry analysis.

CDCA8 expression	Tumor tissue		Normal tissue	
	Cases	Percentage	Cases	Percentage
Low	100	56.8%	24	100%
High	76	43.2%	0	–%

*P* < 0.001.

**Table 2:** Relationship between CDCA8 expression and tumor characteristics in patients with glioma.

Features	No. of patients	CDCA8 expression		*P* value
		Low	High	
All patients	176	100	76	
Age (years)				0.368
≤41	92	55	37	
>41	83	44	39	
Gender				0.983
Male	109	62	47	
Female	67	38	29	
Relapse state				<0.001
Yes	93	39	54	
No	83	61	22	
Grade				<0.001
I	24	19	5	
II	77	52	25	
III	51	24	27	
IV	24	5	19	

### CDCA8 promotes glioma cell proliferation and migration in vitro

**Fig. 2:**
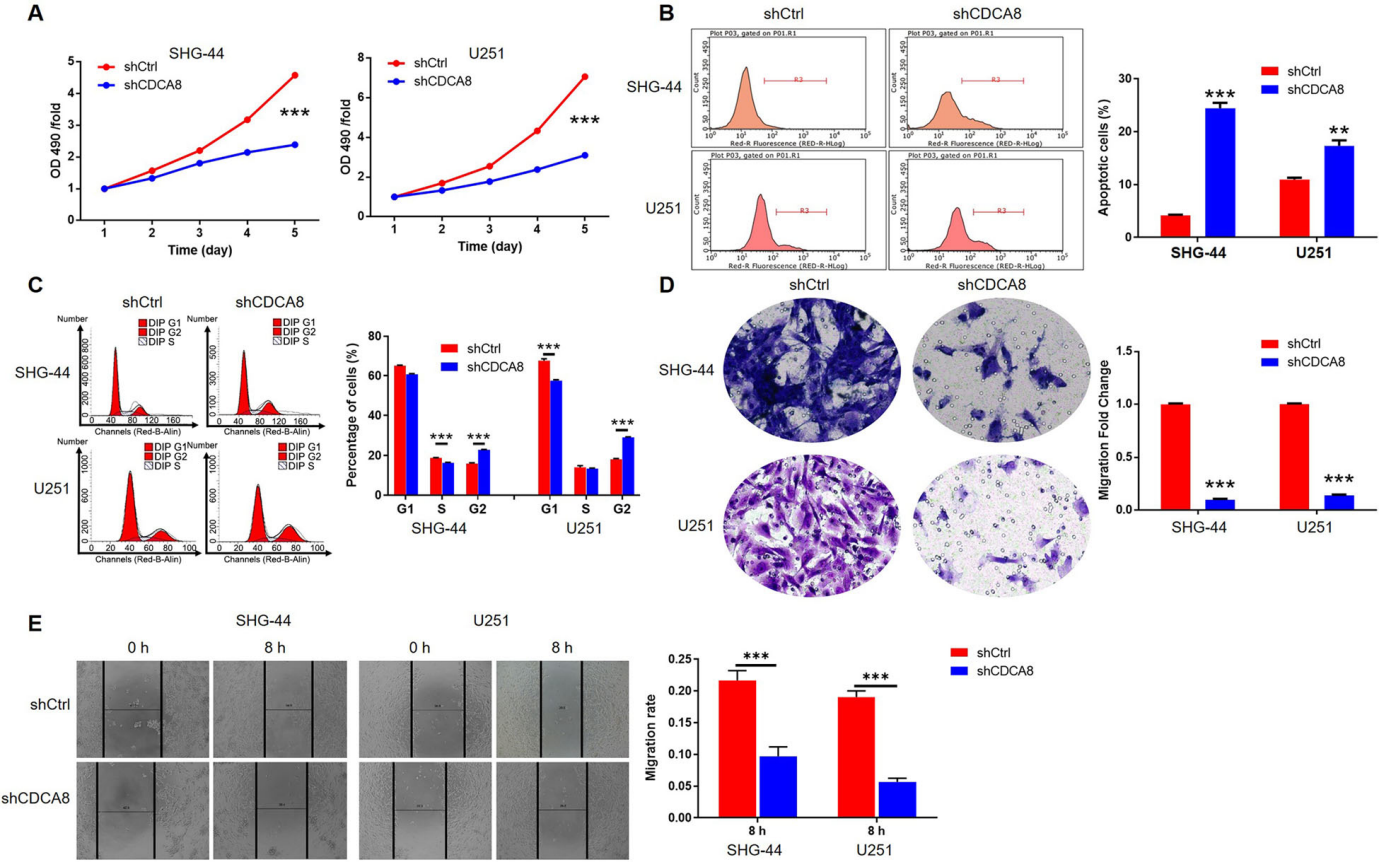
CDCA8 promotes glioma cell proliferation and migration in vitro. **A** The effects of CDCA8 knockdown on SHG-44 and U251 cell proliferation in MTT assay. **B**, **C** Flow cytometry of cell apoptosis (**B**) and cell cycle distribution (**C**) of SHG-44 and U251 cells infected with shCtrl or shCDCA8. **D**, **E** Transwell (**D**) and wound-healing (**E**) assays of CDCA8 knockdown on SHG-44 and U251 cells migration. The experiments were repeated three times independently, and the bars represent SD. **P < 0.01, ***P < 0.001.

To assess the effect of CDCA8 on glioma cell viability, apoptosis, cycle distribution, and migration, we used lentivirus vector to silence the CDCA8 expression in SHG-44 and U251 cells. The derived fluorescence in >80% of cells proved the successful infection (Fig. S1A), and the significantly downregulated level of CDCA8 in SHG-44 and U251 cells (Fig. S1B). As shown in Fig. 2A, B, CDCA8 knockdown inhibited glioma cell proliferation (*P* < 0.001) and induced glioma cell apoptosis (*P* < 0.001). Moreover, knockdown of CDCA8 induced the decreased cell population in S phase and increased in G2 phase (*P* < 0.01, Fig. 2C). We found that anti-apoptosis proteins Bcl-2, Bcl-w, cIAP-2, HSP70, and IGFBP-2 were downregulated, and pro-apoptosis proteins Caspase3, Caspase8, p21, and p27 were upregulated after CDCA8 knockdown (Fig. S2). Furthermore, cell migration was also evaluated by Transwell assays and wound healing, which showed glioma cell migration capacity was suppressed after infected with shCDCA8 (Fig. 2D, E). All these results implied the carcinogenic effect of CDCA8 on glioma progression, which was consistent with our previous results.

### Knockdown of CDCA8 inhibited tumorigenesis and glioma growth in vivo

**Fig. 3:**
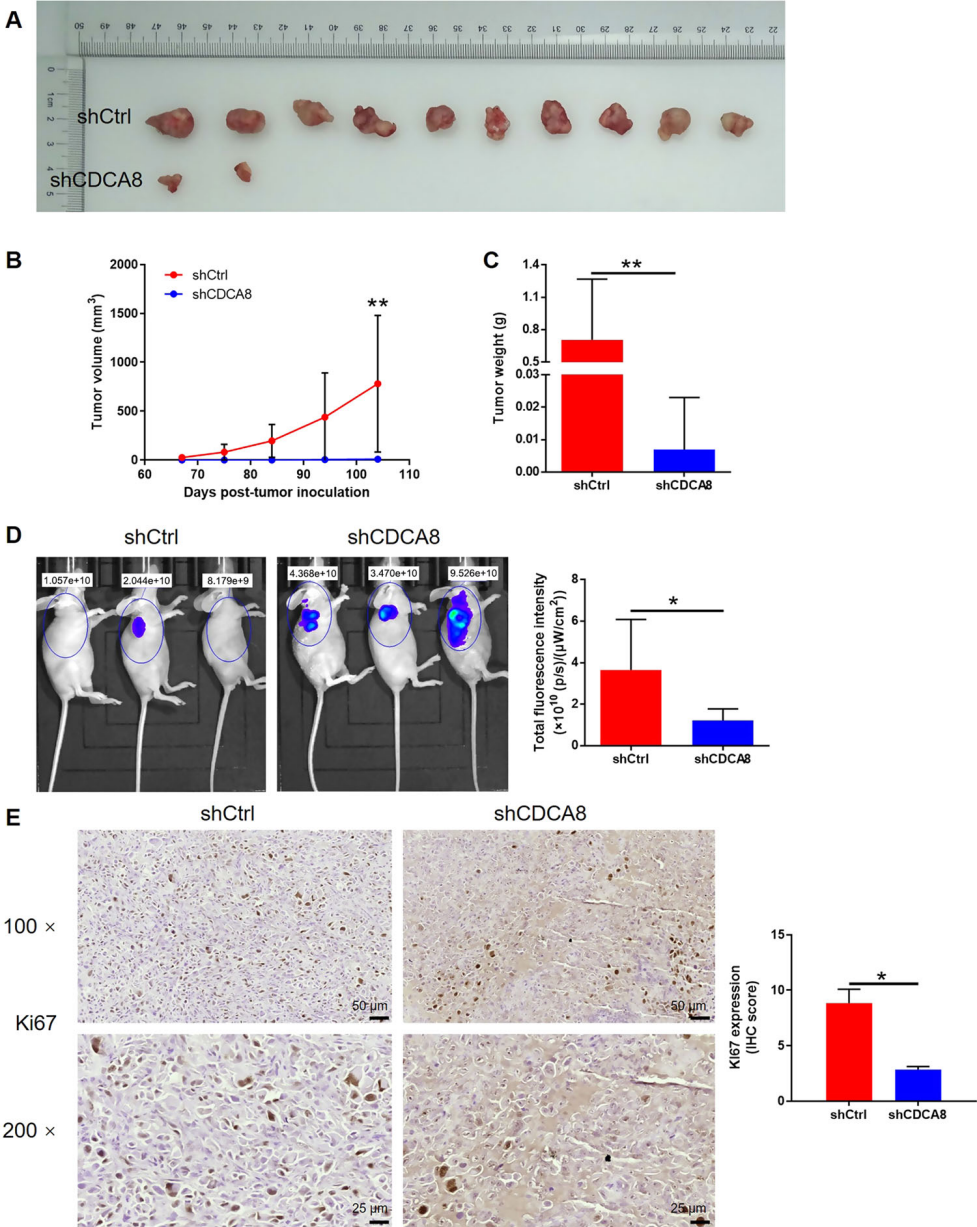
Knockdown of CDCA8 inhibited tumorigenesis and glioma growth in vivo. Mice xenograft models were constructed by subcutaneously injecting U251 cells. **A** Dissected tumors from xenograft models with or without shCDCA8 after implantation were collected for taking photos. **B** Tumor growth curve for tumor volume calculation (from 67 to 104 days post injection). **C** Tumor weight of mice xenograft model analysis was measured after tumor collection. **D** In vivo fluorescence imaging of tumor burden was performed just before sacrificing the mice. **E** Ki67 level by IHC analysis in tumor sections was detected after the tumor collection. Data were performed by mean with SD. *P < 0.05, **P < 0.01.

To investigate the efficacy of CDCA8 knockdown therapy in glioma xenograft model. Interestingly, no obvious tumors were observed in shCDCA8 U251 group, indicating the weak tumorigenesis treated by CDCA8 knockdown (Fig. 3A). The slower growth rate, smaller tumor weight, and lower density of lentivirus vector derived fluorescence formed in shCDCA8 group suggested the restrained progression of glioma in vivo (Fig. 3B–D). Ki67, commonly used as a parameter of proliferative activity of tumors, was detected in all xenografts and found to be downregulated in CDCA8 knockdown group (Fig. 3E). Altogether, CDCA8 knockdown exhibits anti-glioma properties in vivo.

### CDCA8 synergized with E2F1 as the coregulators in glioma

**Fig. 4:**
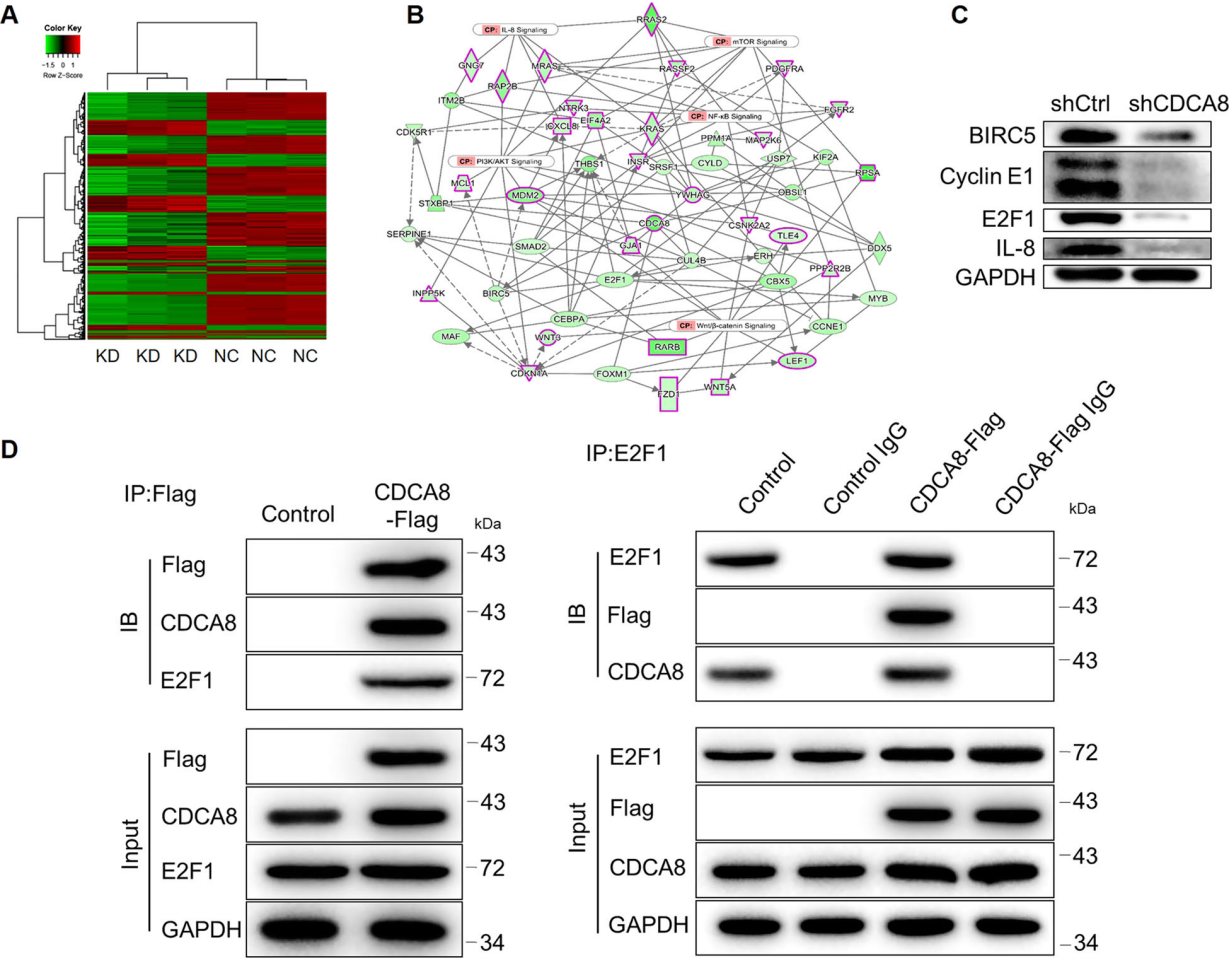
CDCA8 synergized with E2F1 as the coregulators in glioma. The potential genes profile of the microarray analysis in shCtrl and shCDCA8 U251 cells. **A** A heatmap was drawn to show the identified differentially expressed genes between shCtrl and shCDCA8 U251 cells. **B** The CDCA8-related interaction network was constructed based on the enriched canonical signaling pathway. **C** The protein levels of some selected differentially expressed genes (BIRC5, Cyclin E1, E2F1, and IL-8) were evaluated by western blotting. **D** The co-IP assay was carried out in SHG-44 cells for verifying the interaction between CDCA8 and E2F1.

To further explore the underlying mechanism of CDCA8 in glioma progression, a microarray analysis was performed to identify the potential genes regulated by CDCA8. In a total of 299 upregulated and 663 downregulated genes were found following the criteria: log2 fold change >1.3 and FDR < 0.05 (Figs. 4A and S3A). Then, several enriched signaling pathways such as HIPPO and interferon signaling pathways (Figure S3B) and disease and functions such as cancer (Figure S3C) were distinguished to construct the CDCA8-related interaction network (Fig. 4B). Subsequently, several candidates were selected based on the network and subjected further by qPCR and western blotting. As shown in Figs. 4C and S3D, co-expression pattern of CDCA8 and E2F1 was clearly displayed. Moreover, the results of co-IP assay indicated that CDCA8 and E2F1 may act as coregulators in glioma malignancy (Fig. 4D).

### CDCA8/E2F1 axis regulates glioma progression in vitro and in vivo

**Fig. 5:**
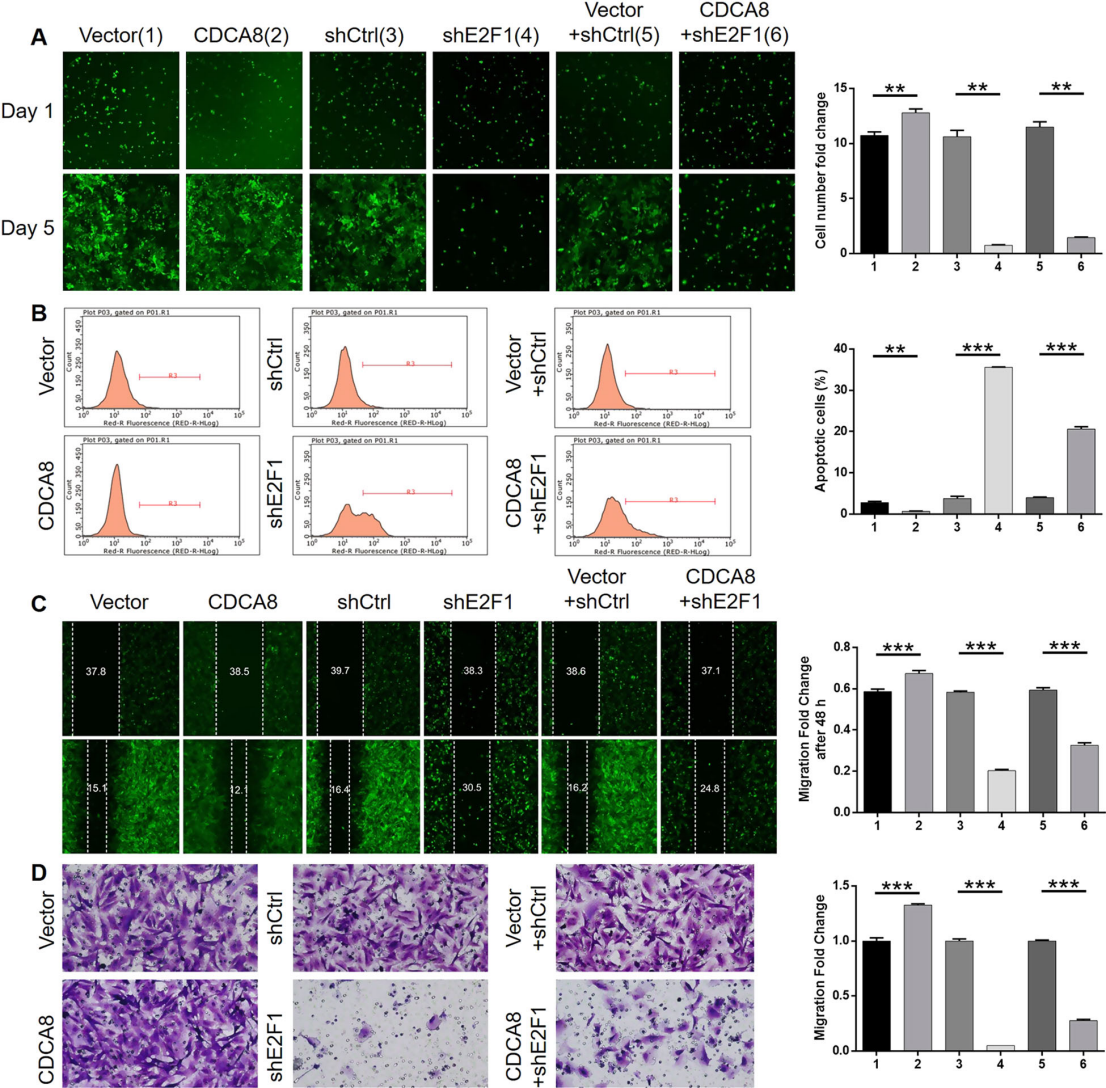
CDCA8/E2F1 axis regulates glioma progression in vitro and in vivo. **A** Celigo cell count assay of overexpressed CDCA8, E2F1 knockdown, or simultaneous CDCA8 overexpression and E2F1 knockdown was used to detect proliferation of U251 cells. **B** The effects of CDCA8/E2F1 on U251 cell apoptosis were assessed by flow cytometry. **C**, **D** The wound-healing (**C**) and Transwell (**D**) assays of CDCA8/E2F1 were used to reveal their regulatory effects on U251 cell migration. The experiments were repeated three times independently, and the bars represent SD. **P < 0.01, ***P < 0.001.

**Fig. 6:**
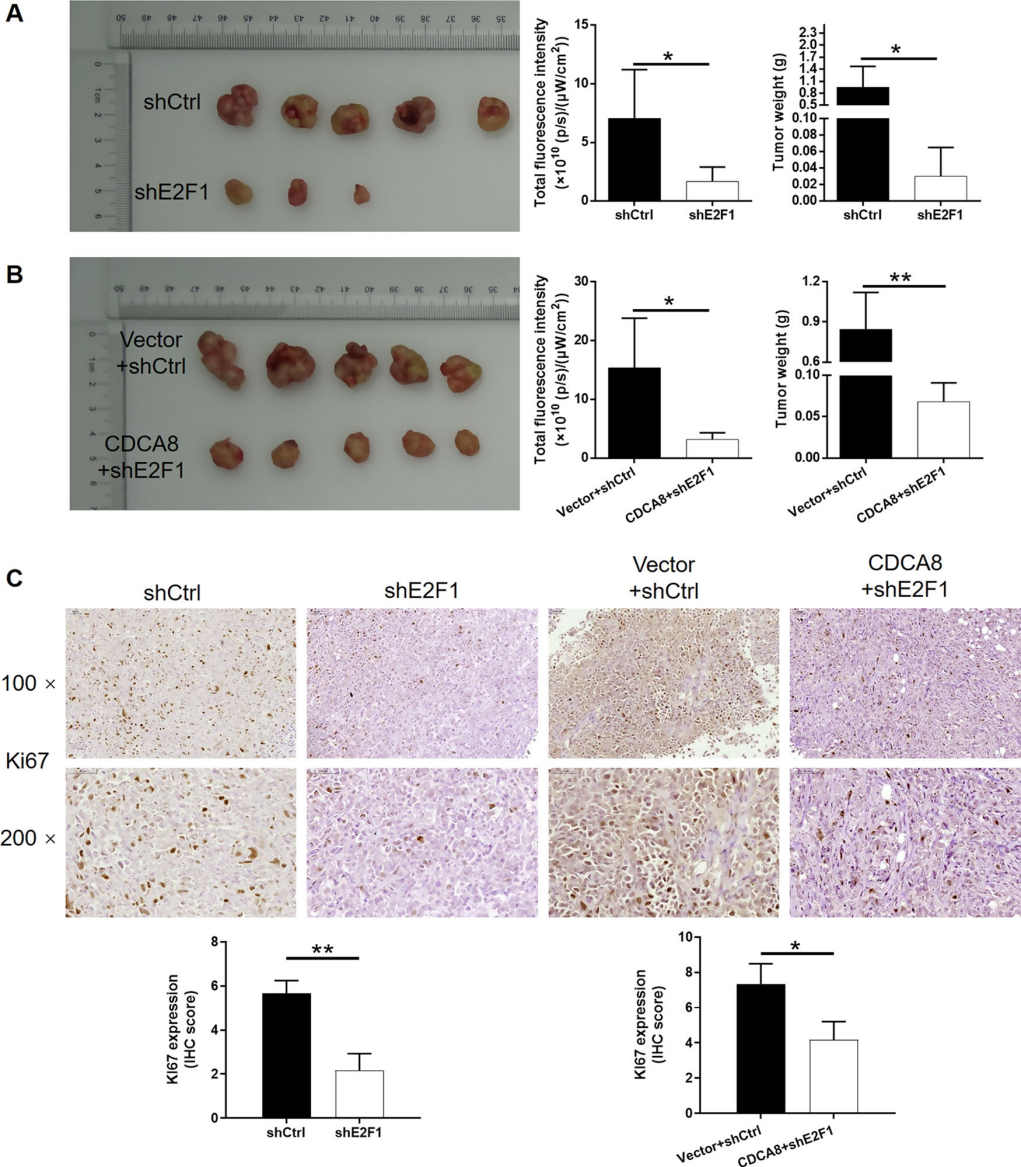
CDCA8/E2F1 axis regulates glioma progression in vitro and in vivo. Mice xenograft models were constructed through subcutaneously injecting U251 cells in shCtrl, shE2F1, Vector+ shCtrl, and CDCA8 + shE2F1 groups. **A**, **B** Dissected tumors from xenograft models with or without shCDCA8 after implantation were collected for taking photos. In vivo fluorescence imaging of tumor burden was performed just before sacrificing the mice. Tumor weight of mice xenograft model analysis was measured after tumor collection. **C** Ki67 level by IHC analysis in tumor sections was detected after the tumor collection. Data were performed by mean with SD. *P < 0.05, **P < 0.01.

To investigate whether CDCA8 regulates glioma development through E2F1, overexpressed CDCA8, and silenced E2F1 lentiviruses were constructed for U251 cells infection. The efficiencies of infection, CDCA8 overexpression, and E2F1 knockdown were shown in Fig. S4, indicating the alleviated CDCA8 overexpression and E2F1 knockdown in CDCA8 + shE2F1 group compared with the single interference group. This phenotype demonstrated that overexpression of CDCA8 promoted glioma cell proliferation (*P* < 0.01, Fig. 5A) and migration (*P* < 0.001, Fig. 5B, C), while inhibiting cell apoptosis (*P* < 0.01, Fig. 5C). Adverse results were obtained in E2F1 knockdown cells (*P* < 0.01, Fig. 5A–D). More than that, outcomes of CDCA8 group, shE2F1 group, and CDCA8 + shE2F1 groups demonstrated that the carcinogenic effect of CDCA8 were generally reversed by simultaneous E2F1 knockdown in vitro and vivo (*P* < 0.01, Fig. 5A–D). Although not statistically significant, it should be observed that CDCA8 promoted tumor growth in vivo (Fig. S5). Subsequently, we further illustrated weaker fluorescence intensity (*P* < 0.05), slower tumor growth (*P* < 0.05), and lighter tumor weight (*P* < 0.05) in both shE2F1 and CDCA8 + shE2F1 groups in comparison with the respective control group (Figs. 6A, B and S6A-B), which was consistent with Ki67 expression in IHC (Fig. 6C). Notably, the inhibition of tumor growth in CDCA8 + shE2F1 group was relatively weaker than that in shE2F1 group. These results indicated the synergistic effects of CDCA8 and E2F1 on the progression of glioma.

## Discussion

Clinically relevant tissue-based biomarkers of gliomas may help neurosurgeons to further classify gliomas with different biological characteristics, improve survival prognosis, and provide therapeutic targets^18,19^. In this study, we demonstrated that high expression of CDCA8 was associated with advanced WHO grade III–IV and poor overall and disease-free survival, indicating CDCA8 may be a biomarker for the high-grade glioma patients’ disease progression and prognosis. Moreover, CDCA8 exhibited its carcinogenetic role in glioma cell growth and migration in vitro and vivo, silence of which could be a promising strategy to suppress glioma progression.

CDCA8 as the main component of CPC is responsible for accurately controlling the normal separation of the cell nucleus and the normal cellular division^20^. CDCA8 is highly expressed in human embryonic stem cells and may have an important role in maintaining stem cell totipotency^21^. Increasing anti-cancer drug researches focus on CPC, the basic regulator, in cell division. Accumulating evidence showed the upregulation of CDCA8 in human cancers and highlighted its biological functions in tumorigenesis. In 2007, Hayama et al. reported the phosphorylation-mediated regulation of CDCA8 by another CPC component Aurora B, thus acting as tumor promotors in human lung cancer^22^. Ci et al. investigated the role of CDCA8 in malignant melanoma, indicating that CDCA8 could promote cell proliferation, migration, and invasion of melanoma cells through suppressing ROCK signaling pathway and predict poor prognosis of patients^23^. Similar CDCA8 functions in the estrogen-stimulated development and progression of breast cancer were also revealed by Bu et al.^24^. Notably, our study is consistent with Bu et al.’s study, which showed CDCA8 regulated apoptosis-related proteins including Bcl-2, p21, and p27^24^. Yu et al. further pointed out that the expression of CDCA8 in breast cancer is associated with tamoxifen resistance^25^. And Dai et al. recognized CDCA8 as a downstream target of transcriptional activation by transcription factor NF-Y in both human embryonic stem cells and cancer cells, demonstrating the potential regulatory mechanism of CDCA8^21^. Besides, abnormal expression and positive regulatory functions of CDCA8 in clear cell renal cell carcinoma and bladder cancer have also been illuminated^26,27^. In our study, we found that CDCA8 promoted glioma cell proliferation by inhibiting cell apoptosis and cell cycle arrest, and enhanced cell migration. Subsequent microarray analysis revealed the potential target genes regulated by CDCA8. Meanwhile, combining with the analysis of CDCA8-related interaction network, we focus on E2F1 as the CDCA8 regulatory protein.

E2F1 is a member of the transcription factors E2Fs family involved in apoptosis and DNA damage response. E2Fs are downstream effectors of the tumor suppressor retinoblastoma gene product pRb and play a role in multiple cytological behaviors such as DNA synthesis and replication, mitotic checkpoint control, DNA damage repair and self-renewal, development, and differentiation^28,29^. And E2F1 is the first discovered and most widely studied gene in the E2Fs family. Existing studies have shown that E2F1 is dysregulated in various types of human cancers including glioma. Zhi et al. described that ECT2 stabilizes and upregulates the expression of E2F1 through interfering with the deubiquitinating ability of PSMD14 in glioma cells^30^. Moreover, E2F1 has been identified as the target molecule of miRNAs in the regulation of development or cisplatin resistance of glioma cells^31,32^. In ceRNAs, one of the most popular molecular regulatory networks, E2F1 is also considered to be involved as a downstream target gene^33,34^. Given E2F1 as oncogene in glioma and our microarray analysis, we deduced that E2F1 may mediate the effect of CDCA8 in glioma. The interaction between CDCA8 and E2F1 verified by co-IP assay showed that CDCA8 may regulate the progression of glioma through interacting and regulating E2F1. As the potential downstream of CDCA8, knockdown of E2F1 could alleviate or reverse the glioma growth induced by CDCA8 overexpression. Thus, CDCA8/E2F1 axis is responsible for glioma cell growth and migration in vitro or in vivo.

In conclusion, despite the limited numbers of clinical specimens and relatively preliminary mechanism research, we defined CDCA8 as a novel tumor promotor in the development of glioma probably through regulating E2F1. CDCA8 is upregulated in glioma and could be considered as a promising target for cancer therapy based on gene silencing. our study reveals a new underlying mechanism of CDCA8 in glioma. We showed that CDCA8 effectively inhibited cell cycle arrest and cell apoptosis in glioma. Synergized with E2F1, CDCA8 promoted glioma cell proliferation and migration in vitro and vivo. Targeting CDCA8 or E2F1 exerted its antitumor effect in malignant glioma. And clinical data showed that CDCA8 may act as a biomarker for the progression and prognosis of glioma. This study provides a novel mechanistic basis and strategy for clinical application of CDCA8/E2F1 in glioma in the future.

## Supplementary information

Figure S1

Figure S2

Figure S3

Figure S4

Figure S5

Figure S6

Table S1

Table S2

Table S3

Table S4

Supplementary figure legends

Table S1

Table S2

Table S3

Table S4
