# Supplementary figures and images for: Double-targeting CDCA8 and E2F1 inhibits the growth and migration of malignant glioma
Source: Cell Death Dis. 2021 Feb 1;12(2):146. doi: 10.1038/s41419-021-03405-4 (PMC7862266; doi:10.1038/s41419-021-03405-4)

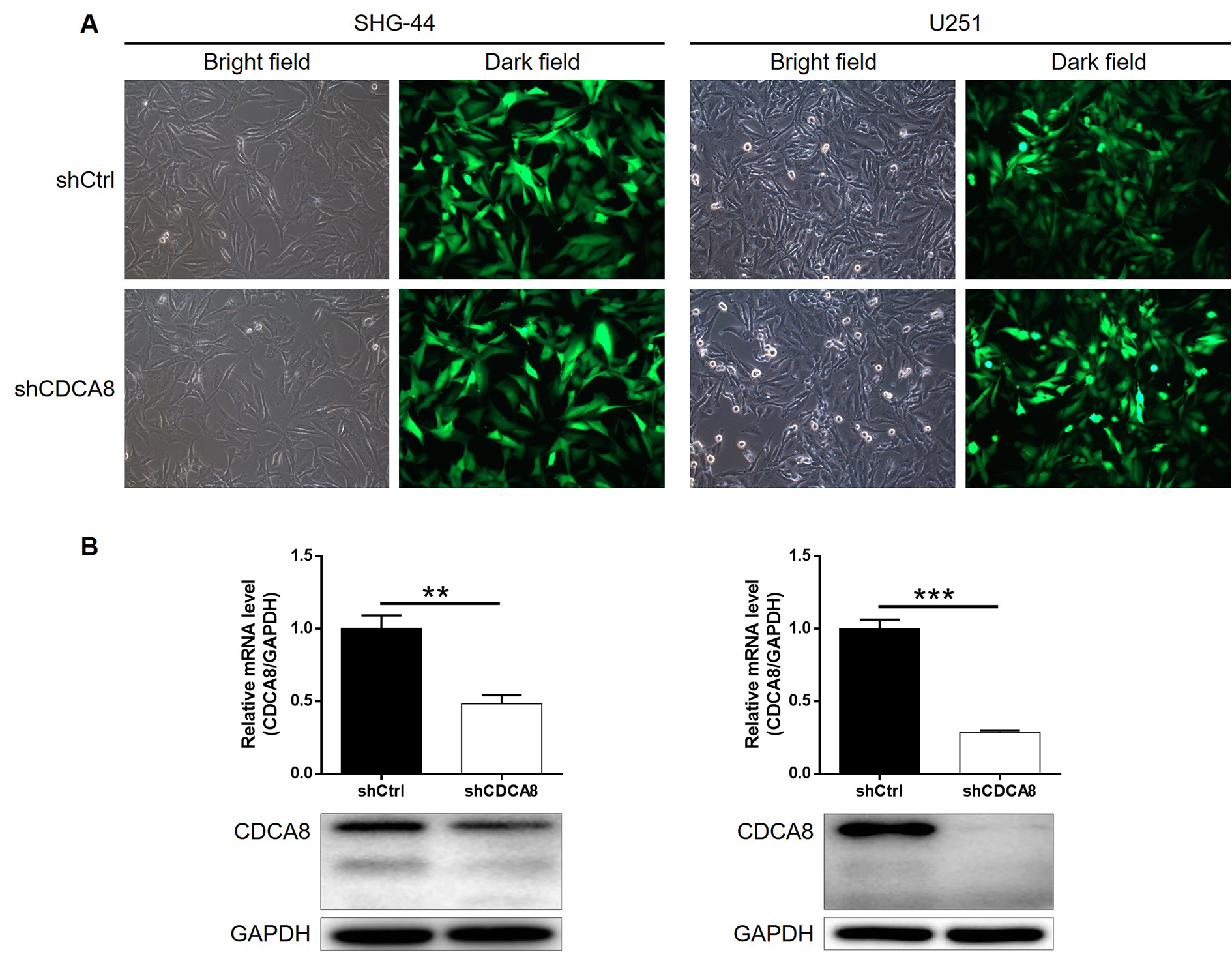

Supplement: Supplementary file 1 — Figure S1 [file 41419_2021_3405_MOESM1_ESM.tif]

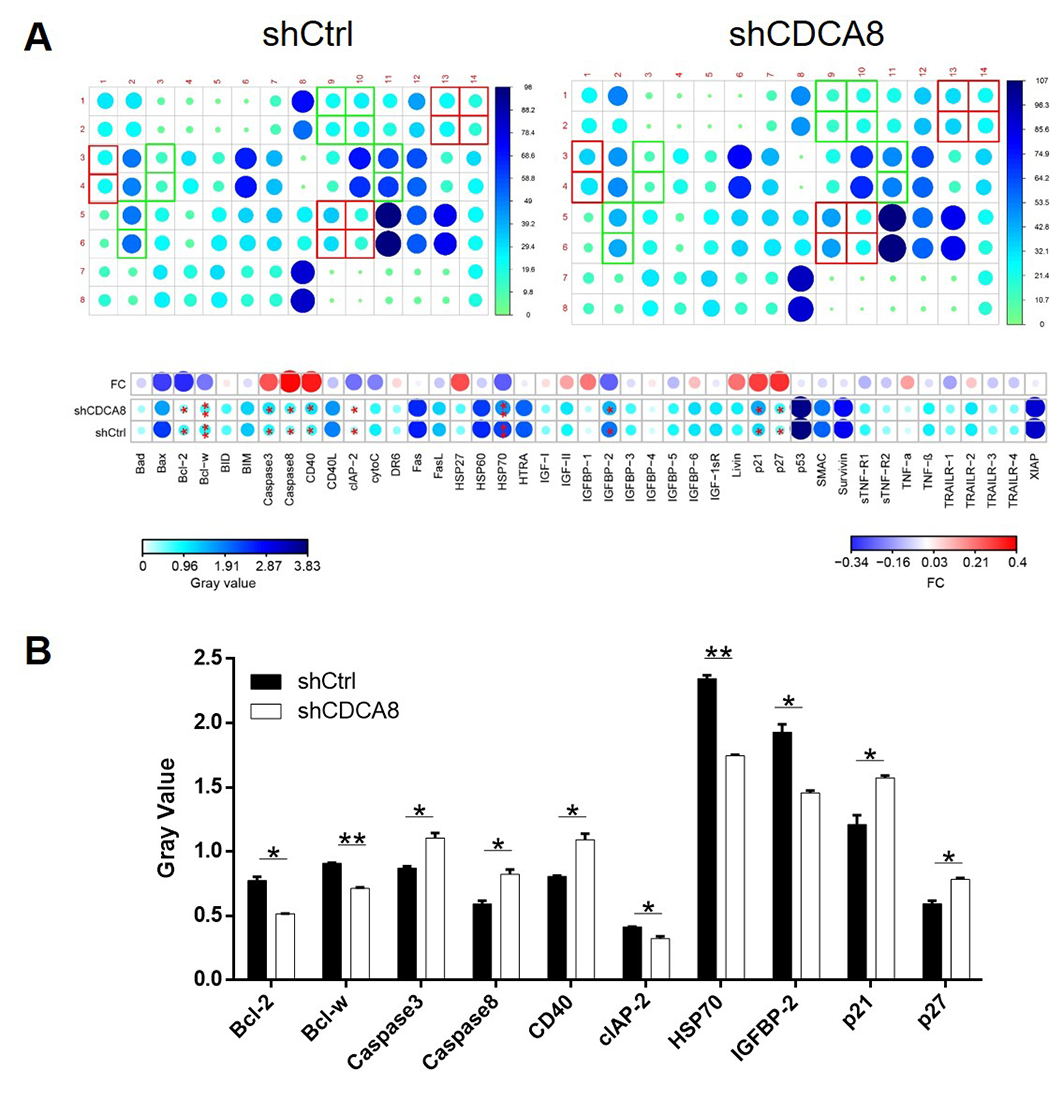

Supplement: Supplementary file 2 — Figure S2 [file 41419_2021_3405_MOESM2_ESM.tif]

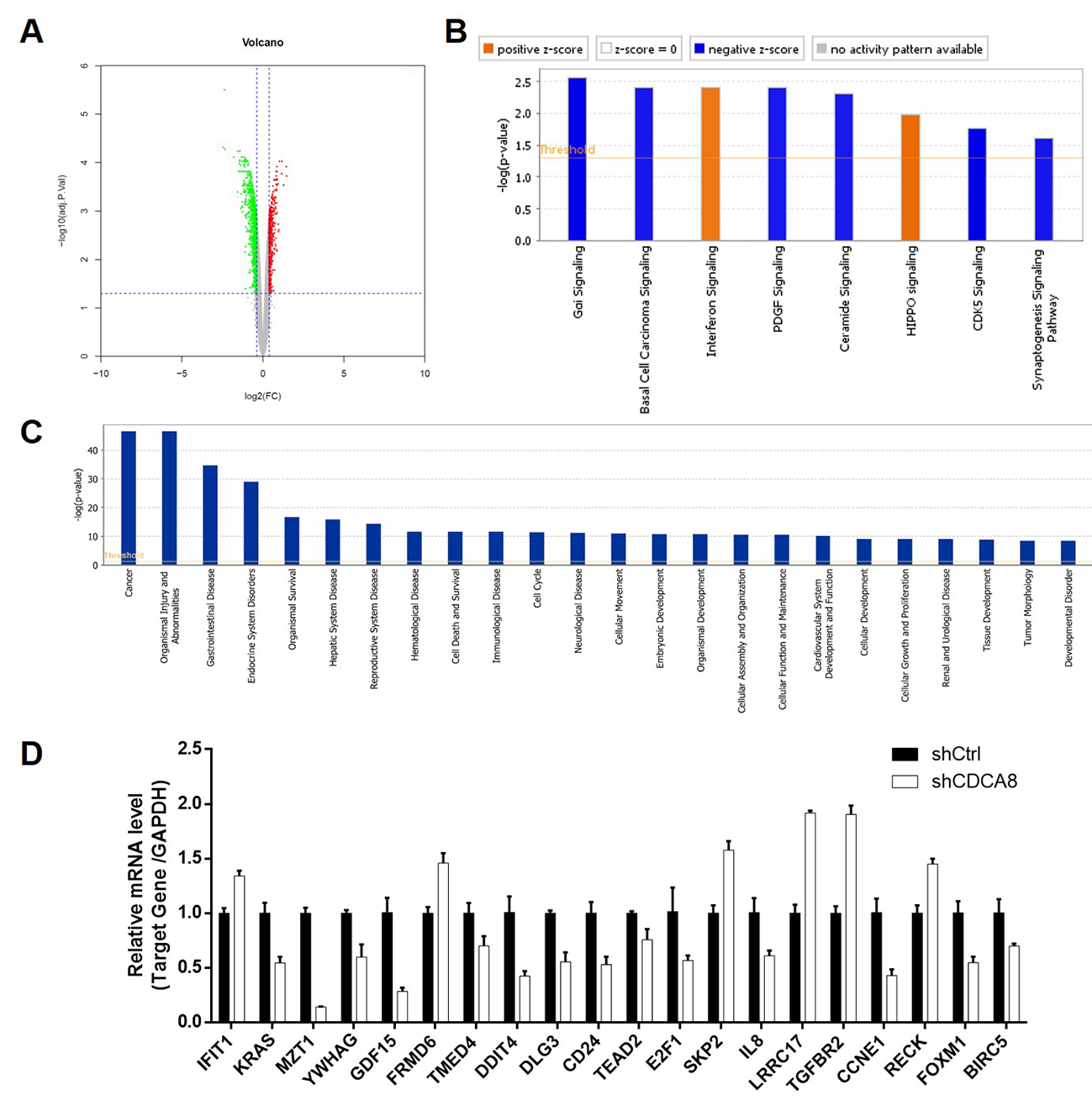

Supplement: Supplementary file 3 — Figure S3 [file 41419_2021_3405_MOESM3_ESM.tif]

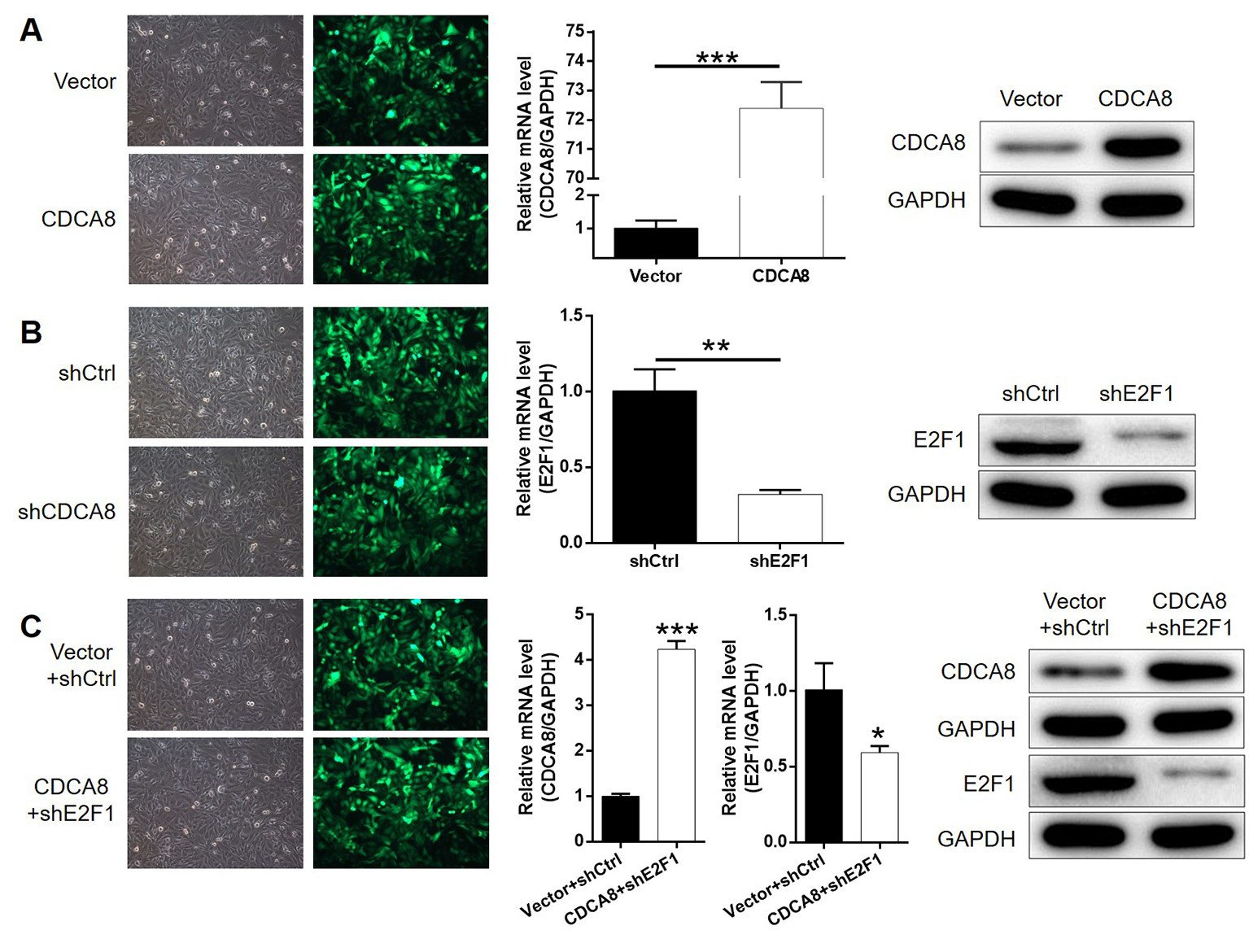

Supplement: Supplementary file 4 — Figure S4 [file 41419_2021_3405_MOESM4_ESM.tif]

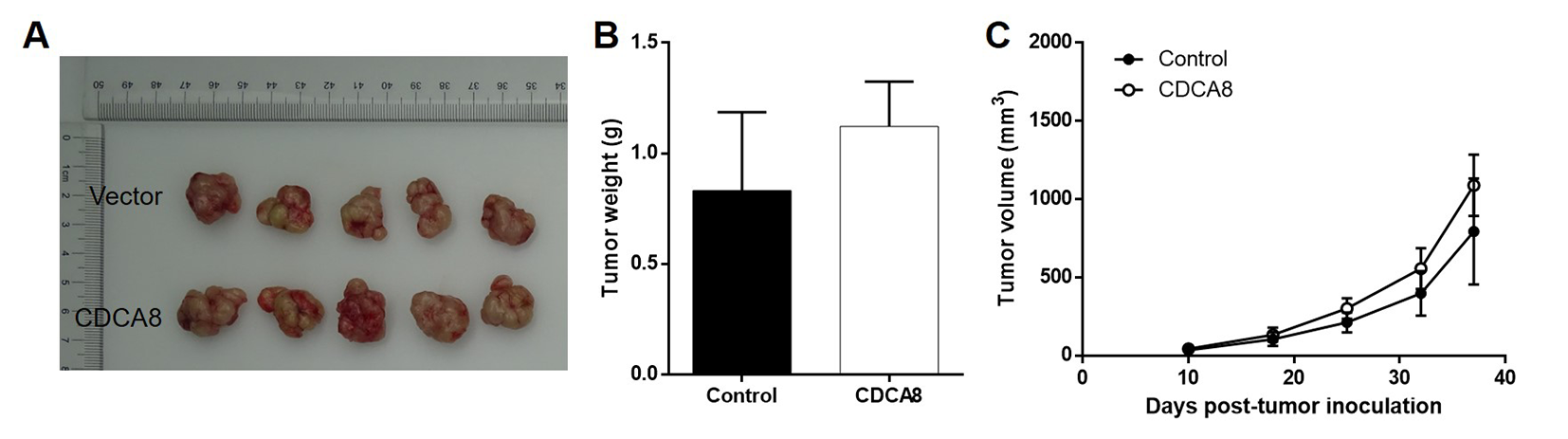

Supplement: Supplementary file 5 — Figure S5 [file 41419_2021_3405_MOESM5_ESM.tif]

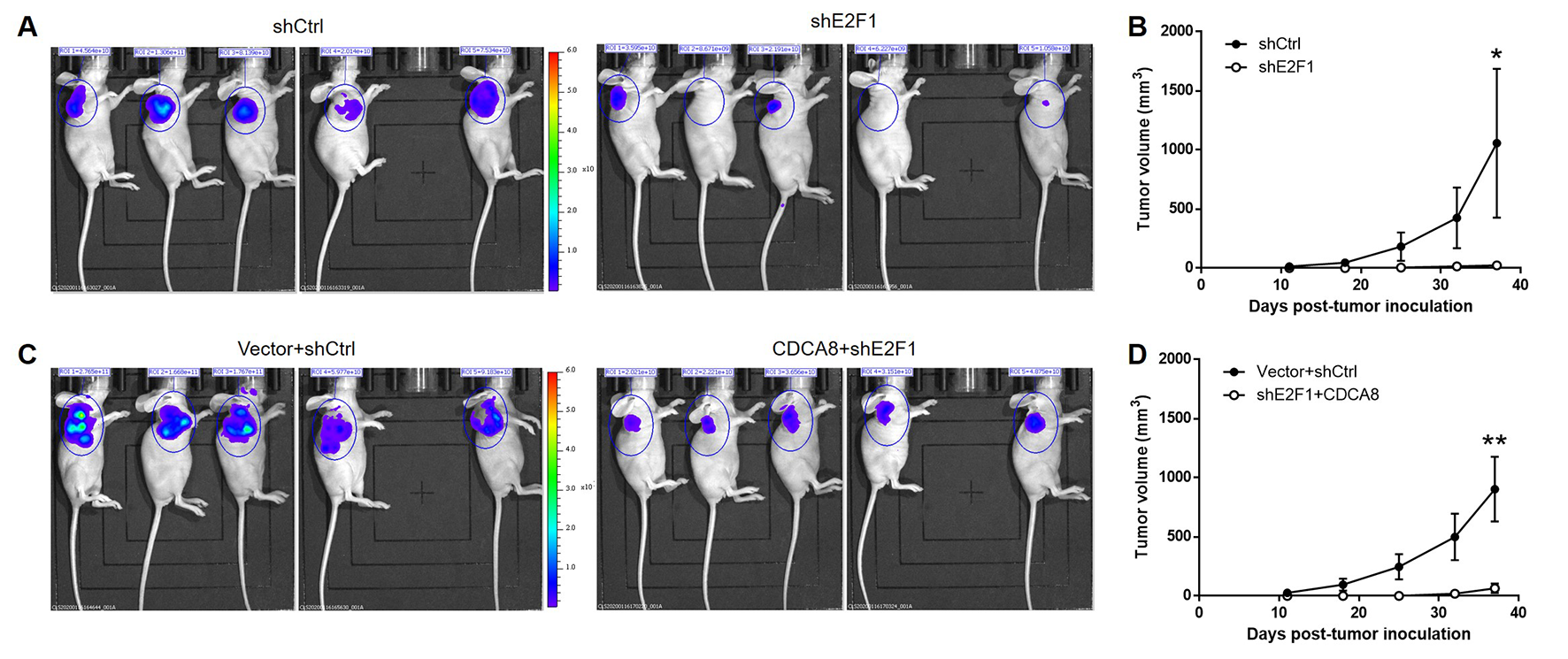

Supplement: Supplementary file 6 — Figure S6 [file 41419_2021_3405_MOESM6_ESM.tif]
