# Supplementary material for: Double-targeting CDCA8 and E2F1 inhibits the growth and migration of malignant glioma
Source: Cell Death Dis. 2021 Feb 1;12(2):146. doi: 10.1038/s41419-021-03405-4 (PMC7862266; doi:10.1038/s41419-021-03405-4)
Supplement: Supplementary file 7 — Table S1 [file 41419_2021_3405_MOESM7_ESM.docx]

Table S1 Antibodies used in western blotting and IHC

| Primary antibodies | Dilution in WB | Source species | Company | Catalog No. |
| --- | --- | --- | --- | --- |
| CDCA8 | 1:1000 | Rabbit | abcam | ab70910 |
| GAPDH | 1:3000 | Rabbit | Bioworld | AP0063 |
| BIRC5 | 1:1000 | Rabbit | Abcam | ab469 |
| Cyclin E1 | 1:500 | Mouse | Abcam | ab3927 |
| E2F1 | 1:1500 | Rabbit | Abcam | ab179445 |
| IL-8 | 1:1000 | Rabbit | Abcam | ab7747 |
| DYKDDDDK tag* | 1:1000 | Rabbit | CST | 14793 |
| * Note: DYKDDDDK tag binds to same epitope as Sigma’s Anti-FLAG M2 Antibody. | | | | |
| Primary antibodies | Dilution in IHC | Source species | Company | Catalog No. |
| CDCA8 | 1:20 | Rabbit | abcam | ab70910 |
| Ki67 | 1:200 | Rabbit | abcam | Ab16667 |
|  |  |  |  |  |
|  |  |  |  |  |
|  |  |  |  |  |
| Secondary antibody | Dilution |  | Company | Catalog No. |
| HRP Goat Anti-Rabbit IgG (WB) | 1:3000 |  | Beyotime | A0208 |
| HRP Goat Anti-Mouse IgG (WB) | 1:3000 |  | Beyotime | A0216 |
| HRP Goat Anti-Rabbit IgG (IHC) | 1:200 |  | Abcam | Ab111909 |
