# Supplementary material for: Double-targeting CDCA8 and E2F1 inhibits the growth and migration of malignant glioma
Source: Cell Death Dis. 2021 Feb 1;12(2):146. doi: 10.1038/s41419-021-03405-4 (PMC7862266; doi:10.1038/s41419-021-03405-4)
Supplement: Supplementary file 8 — Table S2 [file 41419_2021_3405_MOESM8_ESM.docx]

Table S2 The target sequences and shRNA sequences

| Gene | No. | Target sequence (5'-3') | shRNA sequences (5'-3') |
| --- | --- | --- | --- |
| CDCA8 | Pbr10539-a | GTGGAAATACGAATCAAGCAA | CcggGTGGAAATACGAATCAAGCAActcgagTTGCTTGATTCGTATTTCCACTTTTTg |
| CDCA8 | Pbr10539-b | GTGGAAATACGAATCAAGCAA | aattcaaaaaGTGGAAATACGAATCAAGCAActcgagTTGCTTGATTCGTATTTCCAC |
| CDCA8 | Pbr10540-a | GTGGAAATACGAATCAAGCAA | ccggAGATGAAATGATAGTGGAAGActcgagTCTTCCACTATCATTTCATCTtttttg |
| CDCA8 | Pbr10540-b | GTGGAAATACGAATCAAGCAA | aattcaaaaaAGATGAAATGATAGTGGAAGActcgagTCTTCCACTATCATTTCATCT |
| CDCA8 | Pbr10541-a | GCGGAGAGAGCCTGCGATTAT | CcggGCGGAGAGAGCCTGCGATTATctcgagATAATCGCAGGCTCTCTCCGCTTTTTg |
| CDCA8 | Pbr10541-b | GCGGAGAGAGCCTGCGATTAT | aattcaaaaaGCGGAGAGAGCCTGCGATTATctcgagATAATCGCAGGCTCTCTCCGC |
| E2F1 | Pbr10794-a | GGGCATCCAGCTCATTGCCAA | CcggGGGCATCCAGCTCATTGCCAActcgagTTGGCAATGAGCTGGATGCCCTTTTTg |
| E2F1 | Pbr10794-b | GGGCATCCAGCTCATTGCCAA | aattcaaaaaGGGCATCCAGCTCATTGCCAActcgagTTGGCAATGAGCTGGATGCCC |
| E2F1 | Pbr10795-a | CAGCTGGACCACCTGATGAAT | CcggCAGCTGGACCACCTGATGAATctcgagATTCATCAGGTGGTCCAGCTGTTTTTg |
| E2F1 | Pbr10795-b | CAGCTGGACCACCTGATGAAT | aattcaaaaaCAGCTGGACCACCTGATGAATctcgagATTCATCAGGTGGTCCAGCTG |
| E2F1 | Pbr10796-a | GACCTCTTCGACTGTGACTTT | CcggGACCTCTTCGACTGTGACTTTctcgagAAAGTCACAGTCGAAGAGGTCTTTTTg |
| E2F1 | Pbr10796-b | GACCTCTTCGACTGTGACTTT | aattcaaaaaGACCTCTTCGACTGTGACTTTctcgagAAAGTCACAGTCGAAGAGGTC |
|  |  |  |  |
|  |  |  |  |
|  |  |  |  |
|  |  |  |  |
|  |  |  |  |
|  |  |  |  |
|  |  |  |  |
|  |  |  |  |
|  |  |  |  |
|  |  |  |  |
|  |  |  |  |
|  |  |  |  |
|  |  |  |  |
|  |  |  |  |
|  |  |  |  |
|  |  |  |  |
|  |  |  |  |
|  |  |  |  |
|  |  |  |  |
|  |  |  |  |
|  |  |  |  |
|  |  |  |  |
|  |  |  |  |
|  |  |  |  |
|  |  |  |  |
|  |  |  |  |
|  |  |  |  |
|  |  |  |  |
|  |  |  |  |
|  |  |  |  |
|  |  |  |  |
|  |  |  |  |
|  |  |  |  |
|  |  |  |  |
|  |  |  |  |
|  |  |  |  |
|  |  |  |  |
|  |  |  |  |
|  |  |  |  |
|  |  |  |  |
|  |  |  |  |
|  |  |  |  |
|  |  |  |  |
|  |  |  |  |
|  |  |  |  |
|  |  |  |  |
|  |  |  |  |
|  |  |  |  |
