# Supplementary material for: Double-targeting CDCA8 and E2F1 inhibits the growth and migration of malignant glioma
Source: Cell Death Dis. 2021 Feb 1;12(2):146. doi: 10.1038/s41419-021-03405-4 (PMC7862266; doi:10.1038/s41419-021-03405-4)
Supplement: Supplementary file 9 — Table S3 [file 41419_2021_3405_MOESM9_ESM.docx]

Table S3 Primers used in qPCR

| Gene | Forward primer sequence (5’-3’) | Reverse primer sequence (5’-3’) |
| --- | --- | --- |
| IFIT1 | TGAAAAGGTGCTTGAAGTGGAC | GGGGAAGCAAAGAAAATGGC |
| KRAS | GACTGGGGAGGGCTTTCTTT | CTAAGTCCTGAGCCTGTTTTGTG |
| MZT1 | GAGCTTCGCAAGGCTACTGA | CCCTCTTGCAGAGCTTGACATA |
| YWHAG | CTTTGGCTCTGGCTGCTTT | TATCAAGTCTCCCATTCCCTCT |
| GDF15 | GCAAGAACTCAGGACGGTGA | TGGAGTCTTCGGAGTGCAAC |
| FRMD6 | CATCCTGAAGCACATTCCAAAC | TCCAGTCGGACAGCCTCTTT |
| TMED4 | CCAGGGGCTCTACTTCCACA | ACGATAGTTGCCGATGACCA |
| DDIT4 | TTAGCAGTTCTCGCTGACCG | CCAAAGGCTAGGCATGGTGA |
| DLG3 | AGGATGTGAGGCACGAGGAA | TGTCAGCCAAGGCAGTAAAAGT |
| CD24 | TGCTCCTACCCACGCAGATT | GGTGGTGGCATTAGTTGGATTT |
| TEAD2 | AGTGGTGGCTTCTACGGAGTGA | AGAGCAGACCTTGGAGGAACAG |
| E2F1 | CACTTTCGGCCCTTTTGCTC | GTGCTCTCACCGTCCTACAC |
| SKP2 | ATAGAAGTGTCCACCCTCCACG | CACCCAGAAAGGTTAAGTCGC |
| IL8 | CTTGGCAGCCTTCCTGATT | GGTGGAAAGGTTTGGAGTATGT |
| LRRC17 | CCAGATCAAAGTCTTGACGGAG | AAGTACAGTGCCAGGGGTTGTC |
| TGFBR2 | GTGCCAACAACATCAACCACA | GCCTTATAGACCTCAGCAAAGC |
| CCNE1 | CTGGATGTTGACTGCCTTGAA | CGCACCACTGATACCCTGAAA |
| RECK | TGCCAGTTGGGCTGTAGAA | CTCCTTGATCTGACTGTGCATTAC |
| FOXM1 | AGTTCCCGGTGAACCAGTCA | ACACCACCTGTTCCCCAAA |
| BIRC5 | TCTCAAGGACCACCGCATCT | TTTGCATGGGGTCGTCATCT |
| CDCA8 | TTGAGTCAGACAGGCAGAACC | TTCCTCCAAGGGCGAAGTAG |
