# Supplementary material for: Double-targeting CDCA8 and E2F1 inhibits the growth and migration of malignant glioma
Source: Cell Death Dis. 2021 Feb 1;12(2):146. doi: 10.1038/s41419-021-03405-4 (PMC7862266; doi:10.1038/s41419-021-03405-4)
Supplement: Supplementary file 10 — Table S4 [file 41419_2021_3405_MOESM10_ESM.docx]

Table S4 Relationship between CDCA8 expression and tumor characteristics in patients with glioma analyzed by Pearson correlation analysis

| Tumor characteristics | index |  |
| --- | --- | --- |
| Grade | Pearson correlation | 0.351 |
|  | Significance (two tailed) | <0.001 |
|  | n | 176 |
| Relapse state | Pearson correlation | 0.318 |
|  | Significance (two tailed) | <0.001 |
|  | n | 176 |
