# Supplementary material for: Double-targeting CDCA8 and E2F1 inhibits the growth and migration of malignant glioma
Source: Cell Death Dis. 2021 Feb 1;12(2):146. doi: 10.1038/s41419-021-03405-4 (PMC7862266; doi:10.1038/s41419-021-03405-4)
Supplement: Supplementary file 11 — Supplementary figure legends [file 41419_2021_3405_MOESM11_ESM.docx]

**Figure S1.** (A) The efficiencies of shCtrl and shCDCA8 in both SHG-44 and U251 cells were evaluated through fluorescence imaging. (B) The knockdown efficiencies of CDCA8 in SHG-44 and U251 cells were assessed by qPCR and western blotting. Data were shown as Mean ± SD. ** P < 0.01, *** P < 0.001

**Figure S2.** (A) A human apoptosis antibody array was used to detect the expression of 43 apoptosis-related proteins in shCtrl and shCDCA8 U251 cells. (B) The significantly altered proteins were listed based on the analysis of grey value. Data were shown as Mean ± SD. * P < 0.05, ** P < 0.01

**Figure S3.** (A) The volcano plot of the microarray chip detecting the gene expression profiling of U251 cells with or without CDCA8 knockdown. Red dots represent the significantly upregulated genes; green dots represent the significantly downregulated genes. (B) Enrichment of the differentially expressed genes in canonical signaling pathways was analyzed by IPA. (C) Enrichment of differentially expressed genes in diseases and functions was analyzed by IPA. (D) A variety of differentially expressed genes was selected for further verification by qPCR.

**Figure S4.** (A) The infection efficiencies of vector and CDCA8 overexpression plasmids were detected by fluorescence imaging; the overexpression efficiency of CDCA8 in U251 cells were detected by qPCR and western blotting. (B) The infection efficiencies of shCtrl and shE2F1 knockdown plasmids were detected by fluorescence imaging; the knockdown efficiency of E2F1 in U251 cells were detected by qPCR and western blotting. (C) The infection efficiencies of vector+shCtrl and CDCA8+shE2F1 plasmids were detected by fluorescence imaging; qPCR and western blotting were performed to detect the mRNA and protein level of CDCA8 and E2F1 in cells. Data were shown as Mean ± SD. * P < 0.05, ** P < 0.01, *** P < 0.001

**Figure S5.** (A) The photos of xenografts removed from vector or CDCA8 groups were taken after sacrificing mice. (B) The weight of xenografts from CDCA8 group is heavier than that from vector group. (C) The tumor growth rate is higher in CDCA8 group than vector group. Data were shown as Mean ± SD.

**Figure S6.** (A) Tumor burden in shCtrl and shE2F1 groups was assessed through *in vivo* imaging. (B) The calculation of tumor volume showed a higher growth rate in shCtrl group than shE2F1 group. (C) Tumor burden in Vector+shCtrl and CDCA8+shE2F1 groups was assessed through *in vivo* imaging. (D) The calculation of tumor volume showed a higher growth rate in Vector+shCtrl group than CDCA8+shE2F1 group. Data were shown as Mean ± SD. * P < 0.05, ** P < 0.01
